# Interfering With Lipid Raft Association: A Mechanism to Control Influenza Virus Infection By *Sambucus Nigra*

**Published:** 2017

**Authors:** Shahla Shahsavandi, Mohammad Majid Ebrahimi, Ameneh Hasaninejad Farahani

**Affiliations:** a *Razi Vaccine and Serum Research Institute, Agricultural Research Education and Extension Organization, Karaj, Iran.*; b *Biotechnology Department,* *Payame Noor University of East Tehran Tehran, Iran.*

**Keywords:** Influenza virus, Hemagglutinin, *Sambucus nigra*, Lipid raft, Antiviral activity

## Abstract

*Sambucus nigra *(elder) are broadly used species to treat microbial infections. The potential antiviral activity and mechanism action of elder fruit (EF) in human epithelium cell (A549) cultures infected with H9N2 influenza virus were determined. The effect of various concentrations of EF on influenza virus replication was examined by using virus titration, quantitative real time RT-PCR, fusion and lipid raft assays following two treatment procedures: A) pre-treated H9N2 virus with each concentration of EF extract and transfection of A549 cell cultures, and B) each concentrations of EF was added to H9N2 virus infected-cell cultures following virus adsorption. In both treatments with lower doses of EF increased viral titer as well as synthesized viral nucleoprotein as indicating the herb had no inhibitory effects on virus replication. In (B) trial with higher doses, 40 and 80 μg/mL of EF, a significant decrease in virus titer and viral protein synthesis were shown in EF treated cells indicating the herb affect either entry of viruses or inhibition virus particle release. The results suggest that EF treatment of the influenza virus infected-human epithelial cells may involve in lipid raft association which function as platform for formation of viral membrane fusion and budding. Differencesin treatment time and dose of EF extract in infected cells with influenza virus have a marked effect on the efficacy of the herb.

## Introduction

Influenza is a worldwide contagious disease of human and animal populations. The direct transmission of avian influenza viruses to human may raise serious threat to public health and control of pandemic influenza. The H9N2 subtype is high on the list of candidates for next pandemic because of its expanding geographic distribution, circulating in multiple avian species and repeatedly infecting mammals including pigs and humans (1, 2). Vaccination, chemoprophylaxis with specific antiviral drugs, and personal protective non-pharmacological measures are the tools to treat influenza virus infection (3, 4). Two of the viral proteins neuraminidase (NA) and matrix 2 (M2) ion channel are targets for approved anti-influenza drugs (5). The emergence of drug resistant strains of influenza viruses under drug selective pressure (6, 7) highlighted the need to development of new influenza therapies with alternative modes. Inhibition of virus entry, blocking viral replication or blocking virus-supportive signaling processes may be alternative approaches to inhibit virus growth (8-10). Generally influenza viruses ʹentry into target cells mediated by proteolytic cleavage of hemagglutinin (HA) protein through a functional lipid raft cycle then internalizes into host cells through multiple pathways of endocytosis (11). The enveloped viruses require intact cell-surface lipid rafts for efficient cellular entry. The HA sequence contains two raft-targeting signals; a group of acylated cysteines in the cytoplasmic tail, and a group of consecutive hydrophobic residues at the n-terminal end of the transmembrane domain. Palmitoylation of the tail promos fusion pore formation and concentration of HA in membrane rafts provides an efficient fusion activity for cellular membranes (12, 13). It is assumed that lipid rafts as platforms for intracellular sorting and signal transduction events (14) play a decisive role at several steps during virus replication including intracellular transport of viral proteins, assembly and budding of progeny virus at the plasma membrane, environmental stability of the virus particles, and fusion of viral and host cell endosomal membrane upon virus entry (13, 15). Thus, rafts are functionally indispensable for the replication cycle of influenza virus and hence perhaps a possible target for anti-influenza drugs to be developed.

In recent studies a number of herbs have been introduced as potential sources of antiviral drugs (16-23). *Sambucus nigra *(elderberry, elder) is a member of the Caprifoliaceae family, native to Asia, Europe, North Africa and North America. The elder fruit extract was shown to have anti-inflammatory properties due to the anthocyanins cyanidin 3-glucoside and cyanidin 3-sambubioside and to be effective for treating influenza viruses type A and B(24). In this study, if EF has potential anti-influenza virus activity at different concentrations during virus infection of human lung epithelial cells can be controlled was examined. This understanding would help design biochemical compounds with higher potency to serve as potential antiviral for influenza treatment.

## Experimental

*Herbal extract and cytotoxicity assay*

The elderberries were collected from Metkazin valley in Mazandaran province, Iran and dried in open air and shady conditions. The well-dried fruits were ground into fine uniform powder. The aqueous solutions were extracted from 100 g of the dried powders in water bath at 80 °C and kept for 20 min in inert atmosphere then passed through Whatman filter paper. The resulting solution was concentrated using a rotary evaporator. The dried crude concentrated extract was weighed (10.15 g) and stored at 4 °C until use. Five concentrations of the product from 5, 10, 20, 40 and 80 μg/mL were made in distilled water passed through a 0.45 µm filter (Millipore™ membrane filter, USA) and stored at 4 °C until use. Cytotoxicity of the extract was examined by checking cytopathic effect (CPE) and also by cell viability against human lung adenocarcinoma cell line (A549; ATCC CCL-185^tm^). The cells were cultured in Dulbecco’s modified eagle medium (DMEM; Sigma Aldrich) with 10% fetal bovine serum, penicillin 100 U/mL, and streptomycin 10 μg/mL then seeded in 96-well culture plate (approximately 5,000 cells per well). Different concentrations of EF extract were separately added and the plate was incubated for 5 days. The cell viability following treatment with EF substances was determined by 3-(4,5-dimethylthiazol-2-yl)-2,5-diphenyl tetrazolium bromide (MTT, Sigma Aldrich) assay (25).

*Antiviral activity against influenza virus*

An avian influenza H9N2 virus (A/chicken/Iran SS1/1998) was used in this study. The permissivity of A549 cells for the virus has been determined previously (25). The antiviral effect and mechanism of action of EF extract on H9N2 virus were evaluated in two treatment procedures. In (A) procedure the mixture of the virus and each concentration of EF extract were incubated 8 h at room temperature.ThenA549 cell cultures were infected with the pre-treated H9N2 virus at multiplicity of infection (MOI) = 0.1. On (B) trial the five concentrations of EF were separately added to H9N2 virus infected-cell cultures following virus adsorption for 1 h at 37 °C. Up to 72 h post-infection (hpi) and at 16-h intervals, CPE was evaluated microscopically and scored. The reduction in virus replication was calculated as a percentage of the virus control (% virus control = CPEexp/CPEvirus control×100). The 50% inhibitory concentration (IC_50_) was defined as the concentration of substance necessary to reduce virus-induce CPE by 50% relative to a reaction mixture containing virus but no inhibitor and estimated using the Reed-Muench method. In case the substance affected the multiplicity of the virus in other tests and sub culture were performed to screen whether the antiviral acts against virus. The cell viability and virus titer were also determined at 8 h-intervals up to 48 hpi.

**Figure 1:**
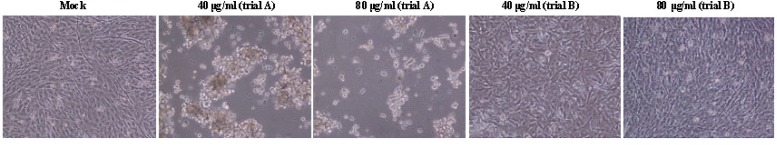
Microscopic analyses of H9N2 influenza virus infected A549 cells after 48 h incubation with different concentrations of *Sambucus nigra *(EF) extract (100X magnification).the influenza-virus infected A549 cells became rounded and eventually detached in (A) treatment with 40 and 80μg/mL of EF in compared with the uninfected cells (mock), while the CPE was not observed in (B) trial

**Figure 2:**
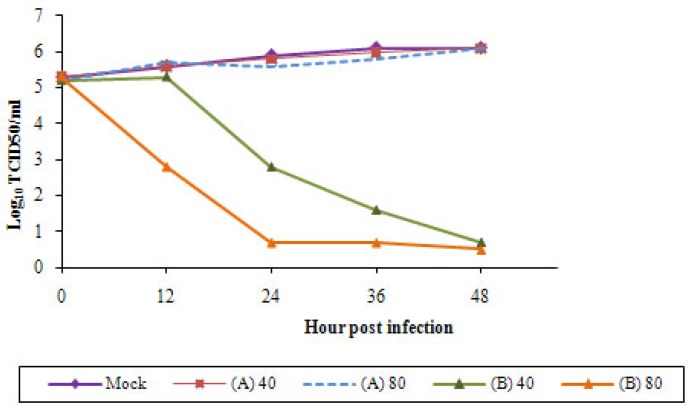
Inhibition of H9N2 influenza virus replication in the presence of different concentrations of *Sambucus nigra *(EF) extract was determined. The titer of virus in A549 supernatants was assayed by TCID_50_ in trials (A) and (B) with 40 and 80 μg/mL of EF compared to the virus-infected and EF-untreated cells (mock

**Figure 3:**
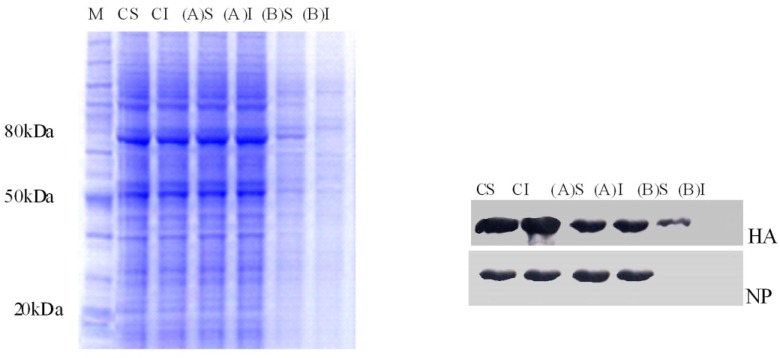
Triton X-100 solubilization analysis of A549cells infected with H9N2influenza virus in pre- and post treatments of *Sambucus nigra *(EF). Equivalent aliquots from soluble material (S) and insoluble (I) fractions of 80 mg/mL of EF in trials (A) and (B) were subjected to SDS-PAGE. Then viral HA and NP were detected by Western blot using specific antibodies. HA incorporated into lipid raft during virus attachment to the cell membrane. In post treatment with the higher dose of EF, the solubility of HA was affected due to the disruption of lipid raft, whereas the solubility of the protein remained unaffected in control (C). The NP floated to the top fraction in (A) treatment

**Table 1:** Nucleoprotein (NP) influenza virus gene expression levels in response to 40 and 80 μg/mL of elder fruit (EF) evaluated by quantitative RT-PCR at the indicated treatment procedures

**Influenza virus NP copy number**										
**Time (h)**	**Mock**	**(A) trial, 40 (μg/mL)**	**(A) trial, 80 (μg/mL)**	**(B) trial, 40 (μg/mL)**	**(B) trial, 80 (μg/mL)**	**β-actin (A), 40 (μg/mL)**	**β-actin (A), 80 (μg/mL)**	**β-actin (B), 40 (μg/mL)**	**β-actin (B), 80 (μg/mL)**	**β-actinmock**
8	26.81 ± 0.23	27.33± 0.31	26.24 ± 0.23	15.37 ± 0.20	10.23 ± 0.22	17.83 ± 0.20	17.90 ± 0.21	17.81 ± 0.20	17.74 ± 0.18	17.75 ± 0.16
16	25.37 ± 0.24	27.06 ± 0.19	26.05 ± 0.23	15.35 ± 0.24	10.20 ± 0.21	17.83 ± 0.20	17.91 ± 0.20	17.72 ± 0.21	17.82 ± 0.21	17.84 ± 0.20
24	24.94 ± 0.27	23.43 ± 0.32	24.92 ± 0.25	15.24 ± 0.20	10.00 ± 0.22	17.91 ± 0.21	17.91 ± 0.21	17.78 ± 0.18	17.76 ± 0.19	17.72 ± 0.19
32	25.37 ± 0.24	27.06 ± 0.19	25.30 ± 0.21	15.25 ± 0.24	10.00 ± 0.21	17.73 ± 0.19	17.86 ± 0.20	17.90 ± 0.17	17.93 ± 0.20	17.76 ± 0.21
40	27.18 ± 0.22	24.12 ± 0.26	26.52 ± 0.24	15.20 ± 0.21	10.00 ± 0.21	17.84 ± 0.21	18.57 ± 0.20	17.84 ± 0.20	18.01 ± 0.21	18.24 ± 0.20
48	27.18 ± 0.21	25.37 ± 0.23	27.04 ± 0.23	15.21 ± 0.20	10.00 ± 0.21	18.12 ± 0.17	18.44 ± 0.21	17.92 ± 0.18	18.22 ± 0.22	18.17± 0.18

*Virus titration*

The virus titer was determined by tissue culture infection dose (TCID_50_) assay. Aliquots of viral supernatants were 10-fold serially diluted with phosphate buffer saline (PBS pH 7.2), applied in A549 cells/well of a 96-well plate, and incubated at 37 °C for 1 h. The inoculum was removed, and the cells were washed with PBS and supplied with DMEM containing bovine serum albumin and trypsin. At the 12-interval hpi, TCID_50_ was analyzed on the basis of the Reed-Muench method.

*Quantitative real time RT-PCR (qRRT-PCR) analysis*

The mRNA levels of viral nucleoprotein (NP) gene from mock and H9N2-infected A549cells in the presence of EF extract were analyzed by one-step qRRT-PCR at 8-hpi intervals up to 48 hpi. Total RNA was extracted using QiaAmp viral RNA mini kit (Qiagen, Germany) and the amount of viral RNA determined to the house keeping gene β-actin as internal control of cellular RNA (25). Reactions were repeated three times for each sample and standard deviations (SD) calculated.

*Fusion assay*

Based on the previous study (26) the fusion activity of ha was evaluated in the H9N2-infected cells were either untreated or treated with EF.

*HA lipid raft solubility assay*

The involvement of virus assembly and budding with membrane rafts are studied by investigating inhibitory effect of virion formation and production on disruption of membrane rafts by cholesterol depletion. The H9N2-infected cells were either untreated or treated with EF at 16 hpi were extracted with 1% triton X-100 (TX-100) in NTE (100 MM NaCl, 10 MM Tris (pH 7.4), 1 MM EDTA) on ice for 30 min followed by centrifugation at 14,000 × g to separate the soluble and insoluble fractions. Both the supernatants and the pellets were adjusted to 1× RIPA buffer (1% deoxycholic acid, 1% TX-100, 0.1% sodium dodecyl sulfate, 10 MM Tris (pH 7.4) plus 0.15 M NaCl prior subjected to SDS-PAGE analysis followed by Western blotting using HA and NP-specific antibodies (Abcam, UK).

*Statistical analysis*

The statistical analysis was performed using SPSS version 15.0.1. *p-*value of <0.05 was considered statistically significant. The results were expressed as mean ± SD for three independent experiments.

## Results

The cellular cytotoxicity of EF on A549 cells was tested at various concentrations. The results showed that the herb was not toxic for A549 cells. The concentration 80 μg/mL of the substance was the threshold dose that did not cause a cytotoxic effect against the cells. The maximum inhibitory effect of EF was to be 5.0 μg/mL. The IC_50 _of EF was measured 70.4 ± 3.2 μg/mL and 68.2 ± 2.5 in (A) and (B) treatments, respectively. The marked CPE include subsequent detachment of cells was evident in influenza-virus infected A549 cells at 48 hpi in (A) treatment with low doses of EF (Figure 1), while the CPE was not observed in (B) trial and the viral replication rate increased up to 48 hpi.

Growth curves of the replicated viruses were examined after infection of A549 cells at MOI=0.1. In (A) trial the EF concentrations did not display inhibitory effect on H9N2 virus. After viral adsorption H9N2 virus was able to replicate in A549 cells in the presence of higher concentrations of EFas in the EF-untreated cells. The infectivity of the influenza virus was not significant (*p *< 0.05) differed in these conditions and the H9N2 virus featured constant titer and infectivity (Figure 2). It is not surprising because in our previous study (26) we have shown that in trypsin-free A549 cells virus replication was clearly delayed. Replication of EF pre-treated viruses in A549 cells indicates that the herb had not antiviral activity. In trial (B) the viral titer was decline in a dose-dependent manner. In treatment with lower concentration of EF the viral titer was highly decreased particularly between 12-24 hpi and no viral titer observed with 80 μg/mL of the herbal extract. Thus, the treatment of the infected-cells with the EF doses affected the titer of the H9N2 virus indicating the substance block entry of viruses to the cells possibly by direct interfering with viral HA. The cell suspensions were cultured two times in A549 cells and no virus was recovered.

The effects of the substance on viral entry into the A549 cells and the difference in the kinetic replication of H9N2 virus between the treatments were examined by qRRT-PCR, fusion activity, and HA raft solubility assay. The release of infectious virus particles into the medium was evaluated in both herbal extract-treated virus-infected and untreated virus infected cells. As listed in Table 1 the results showed a decline in ct value for NP RNA synthesis in H9N2 virus treated by 40 and 80 μg/mL of EF in (B) trial. At the lower concentrations as well as in trial (A) no inhibitory effect was observed which correlated with virus titration results. The average number of fused cell per field in both EF treatments was significantly differed. The number of fused cells was estimated lower in post treatment with higher dose of EF than in other treated cells (12.17 vs 38.06 at average), confirming that the herb inhibited fusogenic activity and the virus entry.

To investigate the effect of EF on HA lipid raft association both TX-100-soluble and -insoluble fractions were immunoprecipitated with the respective antibodies, analyzed by SDS-PAGE, and detected by Western blot. HA protein was insoluble in cold TX-100 in virus-infected and EF-untreated cells (control), (B) trial with low EF doses, and (A) trial procedures. In contrast, for treated cells by80 μg/mL of EF in (B) trial, the HA glycoprotein remained in the soluble fraction (Figure 3). These data indicate that the HA activities of H9N2 can be targeted by EF in A549-infected cells.

## Discussion

Emergence of resistance to the approved drugs drives a necessity for the development of new anti-influenza agents that act at different stages of the viral life cycle with broad-spectrum reactivity against mammalian and bird subtypes. Several studies have been progressed to introduce components to be effective either on entry or replication processes of the virus in host (8-10). A wide variety of different antiviral strategies include inhibition of HA, fusion protein, and RNA polymerase activities and targeting of cellular signaling pathways have been explored in recent years (27, 28). However, some researches focus on the ability of herbs to induce inflammatory mediators as well as possessing antiviral and antioxidant activities (29, 30).

In the present study, we examined the effect of elderberry on lipid raft association of influenza virus in human alveolar cells. Capacity of the human cells to become infected with H9N2 influenza virus was determined by assessment of viral and cellular factors previously (25, 26). The high permissivity of A549 cells for the virus has been related to the expression of TMPRSS, the type II transmembrane serine proteases known to activate viral HA. However, the virus induced apoptosis signaling via mitochondrial pathway in human alveolar epithelial cells, which associated with viral infection in a dose-dependent manner. To assay the safety of EF, its toxicity for A549 cells was evaluated in various dilutions. There was no statistical difference in cells viability when treated with different EF concentrations. The apparent reduction of viral titers with higher doses of EF indicating the herb targets the viral entry process and acts by blocking HA activities not by interfering with virus replication. The viral ha is responsible for entry following sialic acid receptor recognition, attachment and fusion to cell. At the molecular level, association of HA with rafts constitutes part of the signaling machinery necessary for concentrating viral proteins, promoting the production of infectious virus, and budding of virions in polarized epithelial cells (12, 30 and 13). According to the obtained results, primary target for EF is membrane, which inhibits viral HA activities either by involving in formation of membrane fusion or raft association. The less fusion activity in post treatment procedure compared to pre-treatment trial indicated that EF can affect virus-cell fusion mediated by HA. On the other hand, the detection of NP as an internal protein suggests that assembly of virus particles takes place in cells indicating that blockage of virus replication is not the action mechanism of EF against influenza virus. So we were also encouraged to examine the inhibitory effect of EF on viral glycoprotein raft association upon the virus entry into or bud from raft domains. The post treatment results of higher doses of EF indicate that aqueous elder berries extract can inhibit entry of influenza virus by interfering with the association of HA protein with lipid rafts and fusion activity. To evaluate the anti-influenza activity after virus infection, we employed the qRRT-PCR for tracing an internal viral protein. The vigorous expression of NP in pre-treatment and post treatment with low doses of EF revealed that the intracellular viral replication cycle was not impaired. In contrast, the replication of the H9N2 virus was virtually differed by treatment with higher dose of EF. Despite primary replication of virus at 40 μg/mL of EF a dramatically decline in ct value for NP RNA synthesis was seen at 24 hpi, suggesting the newly synthesized virions were not released due to lack of budding. Membrane lipid rafts act as a scaffold of many cellular signal transductions and their involvement in virus entry, assembly, or/and budding process in infection life cycles have been demonstrated (31, 32). The lipid raft is used as a platform to concentrate influenza HA binding receptors thus the virus exploit the signaling capacity of raft domains by mediating efficient fusion. Viral NA is concentrated in rafts for its normal incorporation into virions and budding (12, 13). It has been shown that amino acid residues intransmembrane domain and cytoplasmic tail of the HA protein are important for raft association and fusion activity, respectively. These domains contain palmitoylated cysteine residues that are required for hydrophobic interactions with lipids and cholesterol of membrane rafts (33). Mutations in these sites show reduction in both association with detergent-insoluble glycolipid complexes and promotion of fusion pore formation (34). Transmembrane domains and the cytoplasmic tails of NA are also essential for the association with rafts (33). In this study we show that pre-incubation of H9N2 virus with EF was not inhibit fusion activity of the HA following virus entry. Low concentrations of EF increased infectious virus yield throughout the post treatment period, but higher concentration showed undetectable level or caused a decrease in virus titer at primary incubation time. The results indicate that the herb cannot target influenza virus itself. It seems that EF affects the lipid raft microdomains of plasma membrane due to the inhibition of either fusogenic activity or budding process. According to the committee on herbal medicinal products research (35) the main components of elder fruit are anthocyanins (cyanidin-3-glucoside and cyanidin-3-sambubioside), flavonolglycosids and flavonolester that may contribute to pharmacological activity. It has been demonstrated that anthocyanin possesses potential function in regulating cholesterol distribution in cells (36). Cholesterol promotes the formation of lipid domains that becomes selectively incorporated into the influenza envelope (14) so; depletion of cholesterol leads to disassembly of lipid raft microdomains and dissociation of ha protein bound to the lipid rafts. Because influenza viruses use lipid rafts as their budding platform, dissociation of ha protein bound to the lipid rafts leads to reduction virion budding.

Our study revealed that lipid rafts may play an important role during post-entry stages of influenza virus, since addition of EF following adsorption inhibited virus infection. Incubation of influenza virus infected-A549 cells with higher dose of EF may increase lipid raft integrity and inhibit release of infectious progeny virion particles from infected cells. To determine which of the herb compounds promote raft integrity against influenza virus infection, more examination should be done by labeling the viral HA and raft microdomains so that prevention of raft association and fusion activity of the protein is confirmed.

## References

[1] Butt AM, Siddique S, Idrees M, Tong Y (2010). Avian influenza A (H9N2): computational molecular analysis and phylogenetic characterization of viral surface proteins isolated between 1997 and 2009 from the human population. Virol.J..

[2] Butt KM, Smith GJD, Chen H, Zhang LJ, Leung YH, Xu KM, Lim W, Webster RG, Yuen KY, Peiris JS, Guan Y (2005). Human infection with an avian H9N2 influenza A virus in Hong Kong in 2003. J. Clin. Microbiol..

[3] Moscona A (2008). Medical management of influenza infection. Annu. Rev. Med..

[4] Ong AK, Hayden FG, Enders JF (2007). Antivirals for influenza. J. Infec. Dis..

[5] Boltz DA, Aldridge JR, Webster RG, Govorkova EA (2010). Drugs in development for influenza. Drugs.

[6] Cheung CL, Rayner JM, Smith GJ, Wang P, Naipospos TS, Zhang J, Yuen KY, Webster RG, Peiris JS, Guan Y, Chen H (2006). Distribution of amantadine resistant H5N1 avian influenza variants in Asia. J. Infect. Dis..

[7] Kiso M, Mitamura K, Sakai-Tagawa Y, Shiraishi K, Kawakami C, Kimura K, Hayden FG, Sugaya N, Kawaoka Y (2004). Resistant influenza A viruses in children treated with oseltamivir: descriptive study. Lancet.

[8] Triana-Baltzer GB, Babizki M, Chan MC, Wong AC, Aschenbrenner LM, Campbell ER, Li QX, Chan RW, Peiris JS, Nicholls JM, Fang F (2010). DAS181, a sialidase fusion protein, protects human airway epithelium against influenza virus infection: an in-vitro pharmaco dynamic analysis. J. Antimicro. Chemother..

[9] Furuta Y, Takahashi K, Shiraki K, Sakamoto K, Smee DF, Barnard DL, Gowen BB, Julander JG, Morrey JD (2009). T-705 (favipiravir) and related compounds: novel broad-spectrum inhibitors of RNA viral infections. Antiviral Res..

[10] Malakhov MP, Aschenbrenner LM, Smee DF, Kim DH, Ing A, Campbell ER, Yu M, Fang F (2006). Sialidase fusion protein as a novel broad-spectrum inhibitor of influenza virus infection. Antimicrob. Agents Chemother..

[11] Helenius A (1992). Unpacking the incoming influenza virus. Cell.

[12] Ono A, Freed EO (2005). Role of lipid rafts in virus replication. Adv. Virus Res..

[13] Takeda M, Leser GP, Russell CJ, Lamb RA (2003). Influenza virus hemagglutinin concentrates in lipid raft microdomains for efficient viral fusion. Proc. Natl. Acad. Sci. U.S.A..

[14] Scheiffele P, Rietveld A, Wilk T, Simons K (1999). Influenza viruses select ordered lipid domains during budding from the plasma membrane. J. Biol. Chem..

[15] Leser GP, Lamb RA (2005). Influenza virus assembly and budding in raft-derived microdomains: a quantitative analysis of the surface distribution of HA, NA and M2 proteins. Virology.

[16] Nadim MM, Malik AA, Ahmad J, Bakshi SK (2011). The essential oil composition of Achilleamillefolium L cultivated under tropical condition in India. World J. Agric. Sci..

[17] Park SJ, Kwon HJ, Kim HH, Ryu YB, Chang JS, Cho KO, Rho MC, Park SJ, Lee WS (2010). In-vitro inhibitory activity of Alpinia katsumadai extracts against influenza virus infection and hemagglutination. Virol. J..

[18] Yu C, Yan Y, Wu X, Zhang B, Wang W, Wu Q (2010). Anti-influenza virus effects of the aqueous extract from Moslascabra. J. Ethnopharmacol..

[19] Hudson JB (2009). The use of herbal extracts in the control of influenza. J. Med. Plant Res..

[20] Kuroda K, Sawai R, Shibata T, Gomyou R, Osawa K, Shimizu K (2008). Anti-influenza virus activity of Chaenomelessinensis. J. Ethnopharmacol..

[21] Liu AL, Wang HD, Lee SMY, Wang YT, Du G-H (2008). Structure-activity relationship of flavonoids as influenza virus neuraminidase inhibitors and their in-vitro anti-viral activities. Bioorg. Med. Chem..

[22] Hayashi K, Imanishi N, Kashiwayama Y, Kawano A, Terasawa K, Shimada Y, Ochiai H (2007). Inhibitory effect of cinnam aldehyde, derived from Cinnamomi cortex, on the growth of influenza A/PR/8 virus in-vitro and in-vivo. Antiviral Res..

[23] Wang X, Jia W, Zhao A, Wang X (2006). Anti-influenza agents from plants and traditional Chinese medicine. Phytother. Res..

[24] Zakay-Rones Z, Thom T, Wollan T, Wadstein J (2004). Randomized study of the efficacy and safety of oral elderberry extract in the treatment of influenza A and B virus infections. J. Int. Med. Res..

[25] Shahsavandi S, Ebrahimi MM, Sadeghi K, Mosavi SA, Mohammadi A (2013). Dose- and time-dependent apoptosis induced by avian H9N2 influenza virus in human cells. BioMed. Res. Int..

[26] ZarrinLebas N, Shahsavandi S, Mohammadi A, Ebrahimi MM, Bakhshesh M (2013). Replication efficiency of influenza virus H9N2: a comparative analysis between different origin cell types. Jundishapur J. Microbiol..

[27] Iwai Y, Murakami K, Gomi Y, Hashimoto T, Asakawa Y, Okuno Y, Ishikawa T, Hatakeyama D, Echigo N, Kuzuhara T (2011). Anti-Influenza activity of marchantins, macro cyclic bisbibenzyls contained in Liverworts. PLoS One..

[28] Ludwig S (2009). Targeting cell signalling pathways to fight the flu: towards a paradigm change in anti-influenza therapy. J. Antimicrob. Chemother..

[29] Zhang L, Cheng YX, Liu AL, Wang HD, Wang YL, Du GH (2008). Antioxidant, anti-inflammatory and anti-influenza properties of components from Chaenomelesspeciosa. Molecules.

[30] Sharma M, Anderson SA, Schoop R, Hudson JB (2009). Induction of pro-inflammatory cytokines by respiratory viruses and reversal by standardized Echinacea, a potent antiviral herbal extract. Antiviral Res..

[31] Suzuki T, Suzuki Y (2006). Virus infection and lipid rafts. Biol. Pharm. Bull..

[32] Takahashi T, Suzuki T (2009). Role of membrane rafts in viral infection. Open Dermatol. J..

[33] Takahashi T, Suzuki T (2011). Function of membrane rafts in viral lifecycles and host cellular response. Biochem. Res. Int..

[34] Melikyan GB, Markosyan RM, Roth MG, Cohen FS (2000). A point mutation in the transmembrane domain of the hemagglutinin of influenza virus stabilizes a hemifusion intermediate that can transit to fusion. Mol. Biol. Cel..

[35] European Medicine Agency Science Medicine Health, Assessment report on Sambucusnigra L., fructus EMA/HMPC/44208/2012.

[36] Xia M, Ling W, Zhu H, Wang Q, Ma J, Hou M, Tang Z, Li L, Ye Q (2007). Anthocyanin prevents CD40-activated proinflammatory signaling in endothelial cells by regulating cholesterol distribution. Arterioscler. Thromb. Vasc. Biol..

