# Expression of Concern for Interfering with Lipid Raft Association: A Mechanism to Control Influenza Virus Infection By *Sambucus nigra* [Iran J Pharm Res. 2017; 16 (3): e124953]

**DOI:** 10.5812/ijpr-168991

**Published:** 2025-12-09

**Authors:** Editor-in-Chief IJPR

**Affiliations:** 1 School of Pharmacy, Shahid Beheshti University of Medical Sciences, Tehran, Iran

The editors and publisher of IJ Pharmaceutical Research wish to notify readers of a concern regarding one the articles published in 2017 (1).

Following receipt of a communication from an external whistleblower (Ticket #629402), drawing our attention to a potential issue that has been raised concerning aspects of this article. These concerns relate to the integrity and/or reliability of the data and/or images presented in the publication. At this stage, these concerns have not been confirmed and may be resolvable by a satisfactory explanation or clarification from the authors.

In line with our publication ethics policy and the recommendations of the Committee on Publication Ethics (COPE), we have initiated a formal investigation into these matters. We have contacted the corresponding author to request a detailed response and any supporting documentation that may clarify the issues raised.

While this investigation is ongoing, the conclusions of the article should be interpreted with caution. This Expression of Concern is published to inform readers of the situation and does not, at present, constitute a determination of misconduct or a final judgment on the reliability of the findings.

An update will be provided to our readers as soon as the investigation is complete and a final decision has been reached.

Elham Rezaee

School of Pharmacy, Shahid Beheshti University of Medical Sciences, Tehran, Iran

Editor-in-Chief of IJ Pharmaceutical Research
